# Are Procured Quantities of Implants Adequate and Appropriate? Modeling Procurement, Inventory, and Consumption of Contraceptive Implants During Rapid Uptake

**DOI:** 10.9745/GHSP-D-19-00017

**Published:** 2019-06-24

**Authors:** Laila Akhlaghi, Alexis Heaton, Yasmin Chandani

**Affiliations:** a John Snow, Inc., Arlington, VA, USA.; b inSupply Health LTD, Nairobi, Kenya.

## Abstract

Donors and others are concerned that implants procured under the Family Planning 2020 Initiative exceed the number sought by clients, resulting in accumulating stocks. To explore this issue, we examined 3 questions across 9 countries: (1) How accurate were procurement quantities given requirements for filling supply chains for the rapidly growing implant programs? (2) Is there a standard factor that can be applied to consumption data to predict procurement volumes required? (3) How accurately do demographic estimates mirror dispensed-to-client data? We created a model incorporating public-sector supply chain system parameters to calculate system “imputed” inventory and the system “filled-to-max” inventory. Comparing results determined the adequacy of the procurement quantities. The proportion of consumption that the filled-to-max inventory represented through time suggests whether a standard factor can be applied to consumption to predict necessary procurement volumes. We compared demographic estimates to consumption data to determine the usability of the former in predicting demand. According to model results, 3 of the 9 countries came close to procuring accurate quantities over the study period between 2010 and 2017, 4 had procurement volumes lower than what was required to fill the supply chain to maximum inventory requirement levels, and 2 had volumes that exceeded the need. We found no standard factor for relating inventory quantities to consumption rates across countries, given that inventory needs can vary based on system design parameters and the rates of growth or decline in consumption. Finally, we observed that our demographic estimates were on average lower than the dispensed-to-client data in the 6 countries for which these data were available. Study results show that the significant investments in procurement quantities for the rapidly growing implant programs were justified based on consumption and system design. This research should assure observers that rapid increases in implant procurement quantities (where data are available) have generally not resulted in overstocks of the system to date. It suggests that the relationship between procurement quantities and consumption levels cannot be accurately assessed without understanding the country supply chain, inventory control parameters, and current and future demand.

## Abstract (precis)

Recent rapid increases in implant procurement have not resulted in system overstocks to date. We found no standard factor for relating inventory quantities to consumption rates. Rather, that relationship requires specific understanding of the country supply chain, inventory control parameters, and current and future demand.

## BACKGROUND

The Family Planning 2020 (FP2020) initiative was launched in 2012 in recognition of high unmet need for contraception, especially in low- and middle-income countries. FP2020 set a target of reaching an additional 120 million women with contraception by the year 2020.^1^ An important part of the strategy to achieve the goal of increased modern contraceptive prevalence rate (mCPR) has been increasing access to long-acting reversible contraceptives, specifically hormonal contraceptive implants.

Despite their high level of effectiveness, initial uptake of contraceptive implants was low, due to the high upfront purchase price of products and barriers associated with introduction.^2^^,^^3^ Recognizing these limitations, a consortium of public and private organizations―the Bill & Melinda Gates Foundation; the Clinton Health Access Initiative; the governments of Norway, Sweden, the United Kingdom, and the United States; and the Children's Investment Fund Foundation, with support from the United Nations Population Fund―collaborated to establish the Implants Access Program. Through this program, Bayer Pharma's Jadelle and Merck/MSD's Implanon and Implanon NXT were made available to women in the world's poorest countries, as recognized by FP2020,^1^ through price reductions of approximately 50%^4^ from 2013 through 2018, backed by volume guarantees ensuring that minimum quantities would be purchased each year.

As a result of the price reductions and efforts to increase accessibility via more trained service providers, procurements of contraceptive implants in the FP2020 markets and their consumption have risen substantially since 2013.^5^^,^^6^ Implants as a proportion of total mCPR continue to increase in many countries, as measured by surveys such as Demographic and Health Surveys (DHS) and Performance Monitoring and Accountability 2020 (PMA2020).^7^^,^^8^

The rapid uptake of implants combined with limited data visibility into consumption in many countries generated questions from donors, procurers, and other partners about the relationship between product volumes procured and product volumes consumed—specifically, they wanted to know whether increases in procurement quantities were resulting in increases in implant use. Initial review of procurement quantities and demographic surveys, especially in a number of the high-volume procuring countries, led to concerns that procurement quantities far exceeded the number of women seeking this method and were resulting in stocks accumulating in countries,^9^ and the objectives of the Implants Access Program were consequently not being met.

There were concerns that procurement exceeded demand for implants and the Implants Access Program objective of increasing use of implants was not being met.

While it was hypothesized that some of the excess quantities were procured for inventory, the lack of access at the global level to country supply chain data (logistics management information systems) meant that this hypothesis could not be studied effectively across countries using readily available survey statistics. To further explore the relationship between procurement volumes and consumption, we reviewed logistics and demographic data from 9 countries to understand 3 questions:

1. How accurate were procurement quantities given requirements for filling supply chains for the rapidly growing implant programs?
2. Is there a standard factor that can be applied to consumption data to predict procurement volumes required?
3. How accurately do demographic estimates mirror dispensed-to-client data?

## METHODS

**FIGURE 1:**
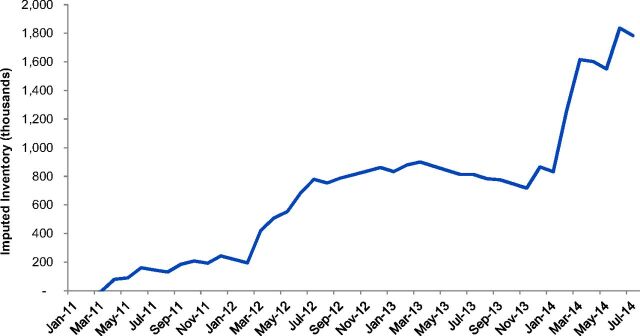
Imputed Inventory, Ethiopia Model as an Example

In order to determine whether procurement quantities were accurate for the inventory needs of countries, we created an Excel-based model whereby we could input supply chain system parameters as defined by the country's supply chain design, historic procurements (quantities entering the supply chain), and estimated consumption and/or implants dispensed to clients (quantities leaving the supply chain via patient use). The model used these data to calculate the imputed inventory for each month of the review period. Figure 1 depicts the imputed inventory for the country example of Ethiopia, and the details on the calculation steps are provided in Supplement 1.

**FIGURE 2:**
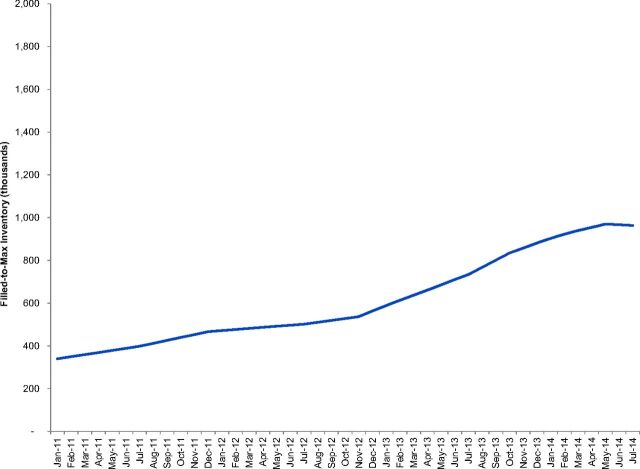
Calculated Filled to Max Inventory, Ethiopia Model as an Example

Using the same model, we built a second scenario using the same estimates of consumption, but instead determined the system filled-to-max inventory required to fill the system to *maximum* stock level for a given country's supply chain design. Figure 2 depicts the filled-to-max inventory for Ethiopia as an example.

**FIGURE 3:**
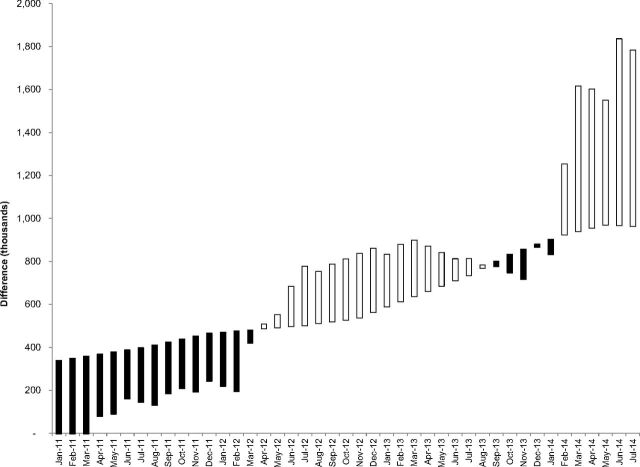
Deficit and Surplus of System Imputed Inventory Compared With System Inventory Filled to Max White bars represent surplus; black bars represent deficit.

A comparison of the 2 results (imputed and filled-to-max inventory) shows either a surplus or deficit for each month of the model as shown in Figure 3.

Using the following equation, we could determine the percentage difference for each month (formula below) and answer our first question about accuracy of procurement quantities.

$$\frac{imputed\;inventory-filled-to-\max\;inventory}{filled-to-\max\;inventory}=Percentage\;difference$$

The result gives us an estimate of procurement quantity accuracy by month. If there was no difference between the imputed and the filled-to-max inventory quantities (i.e., a percentage difference of 0%), procurement quantities for the system were deemed to be accurate and appropriate for that month. If the percentage difference was above 0%, it would indicate over-procurement, and if the percentage difference was below 0%, the indication would be under-procurement.

With the results of the filled-to-max inventory, we could then calculate the proportion of the consumption that the filled-to-max inventory represented through time. The resulting ratio answered our second question about a standard factor. Calculation steps for answering the second research question are included in Supplement 2.

Because we collected 2 different inputs for demand or consumption, the first being dispensed-to-client data reported by information systems in country (where available) and the second estimated using demographic data, the 2 were compared to determine their relationship and answer our third question about the accuracy of demographic estimates.

As part of the model design, we chose to input the data and capture outputs by month through the review period rather than use annual aggregates and averages. Most of our inputs were already available as monthly figures such as those for dispensed-to-client data and procurements. In addition, multiple PMA2020 reports may exist per given year. We also assumed that monthly results would better align with actual movements in the supply chains because reporting and distribution in many settings is done on a monthly, bimonthly, quarterly, or other periodic basis depending on the level of the supply chain. The only input data not available on a monthly schedule were survey results, so best efforts were made to use midpoint months when analyzing demographic surveys instead of publication dates. Linear interpolation was used to determine monthly estimates between points.

Finally, we evaluated the model outputs of imputed inventory by comparing the model results with actual collected and reported system inventory from 1 country, as discussed in more detail in the Model Evaluation section.

We evaluated the model accuracy by comparing its imputed results with collected and reported system inventory for 1 country.

### Defining Procurement, Inventory, and Supply Chain Parameters

For most FP2020 countries, the national supply chain for imported contraceptives, including implants, typically begins at the port of entry. Once products clear customs, they are typically received at the central warehouse, distributed to regional and/or district distribution points (and potentially other sublevels), and used to resupply health facilities, commonly known as service delivery points (SDPs). In this article, we refer to this flow as the in-country supply chain. Contraceptives need to flow through each level of this supply chain to be available as inventory at the SDP as a choice for a woman seeking contraception on any given day. The more levels in an in-country supply chain, the longer the time needed for products to reach the SDP and the further in advance they have to be procured.

Countries determine procurement quantities based on supply (or procurement) plans using forecasted demand. Forecasts should *ideally* be based on historic demand and growth patterns, with demographic parameters being a less ideal foundation or an alternative method used for comparison.^10^ Adjustments to forecasts can be made based on market intelligence of trends and/or other factors assumed to affect future demand (such as budgets, goals, trainings, and/or policies). Supply plans determine quantities procured by taking into account this forecasted demand, available inventory, inventory control policies at each level of the supply chain (such as maximum and minimum stocking parameters), storage capacity, and potential loss/expiry.

Holding inventory at each level of the supply chain is critical to buffer against uncertainty of demand (demand variability), as forecasts are often inaccurate.^10^^–^^12^ Additional inventory (or safety stock) is particularly important for a product with increasing consumption, such as contraceptive implants,^6^ for which it is difficult to anticipate rapid changes in demand. Additional inventory (shelf-life allowing) is also especially critical in the public sector supply chains for many of the countries in the FP2020 market where procurements may happen only once or twice a year and the ability to respond quickly to sudden changes in demand or resources is limited.

To ensure sufficient quantities of products when and where they are needed, the entire supply chain should be filled with inventory in advance of demand and refilled routinely. As products are given to clients or distributed from one point to another, inventory held at higher levels will flow down to the next level (under most demand variabilities) to make sure that product is available to meet future client demand at SDPs. Ensuring that sufficient inventory is available at all levels of the supply chain requires accurate forecasts, routine and accurate data on stock from all levels of the system, and timely procurements. As part of supply chain design, supply chain systems establish inventory control parameters to determine how much of a product needs to be held at each level based on frequency and lead time for resupply. Typically, the inventory control parameters are reflected in a relative measure of months of stock and use current and projected consumption levels per month.

Ensuring that sufficient inventory is available requires accurate forecasts, routine and accurate data on stock, and timely procurements.

**FIGURE 4:**
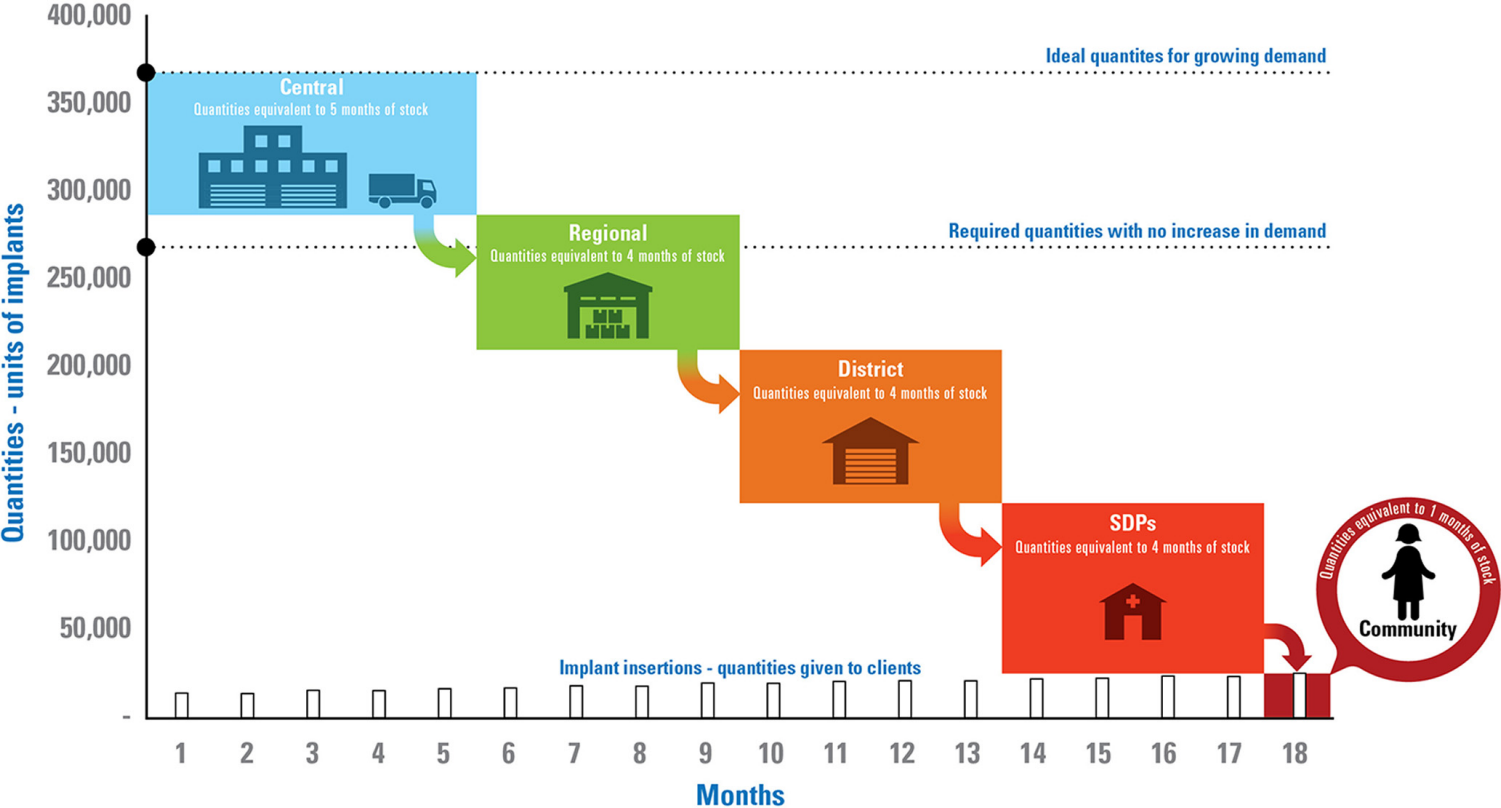
Pictorial Depiction of Maximum Inventory Needs (System and Level) and Flow Through In-Country Supply Chain With Growing Demand in a Sample Country

For example, a system that requires 18 months of stock to fill the supply chain to maximum system levels (e.g., 5 months at central level + 4 months at regional level + 4 months at district level + 4 months at SDP + 1 month for the community health worker) and using 15,000 units a month would require about 270,000 implants to meet client demand. For a similar system that sees demand increase about 75% from month 1 to month 18 (15,000 units in month 1 to more than 26,000 units in month 18)would require 370,000 units (100,000 more units for the full period of months 1–18). A waterfall graph (Figure 4) depicts the quantity needed to be procured (to enter the system) and flow through the various distribution points to reach the point of consumption.

### Data

To use the model developed to determine if procurement quantities are adequate and appropriate to meet the demand of women choosing implants, we reviewed data available from 9 countries (Burkina Faso, Ethiopia, Ghana, Kenya, Pakistan, Tanzania, Uganda, Country A, and Country B). Country selections were based on availability of (1) procurement data (described further below); (2) demographic data (at least 2 time points), listed in the Appendix, used to estimate users of implants; (3) a reliable source of dispensed-to-client consumption (Table 1), a second alternative figure for estimating users of implants; and (4) information on the in-country supply chain design (Table 2). Two of the 9 countries have been anonymized (Country A and Country B) to enable inclusion of their data in this article.

**TABLE 1.** Source and Dates for Distribution-to-Client Data

Country	Source of Direct-to-Client Distribution of Implants	Period Covering Direct-to-Client Distribution Data	Period Using Demographic Estimates Instead of Distribution-to-Client Data
Burkina Faso	PipeLine database	January 2010–June 2017	n/a
Ethiopia	n/a	n/a	January 2011–December 2017
Ghana	DHIMS	January 2010–December 2010 and	January 2011–December 2011
		January 2012–December 2017	
Kenya	DHIS 2	January 2012–December 2017	January 2010–December 2011
Pakistan	eLMIS	January 2010–December 2017	n/a
Tanzania	eLMIS	Aug 2013–December 2017	January 2011–July 2013
Uganda	n/a	n/a	January 2010–December 2017
Country A^a^	Annual Statistical Survey	September 2014–December 2017	January 2010–August 2014
Country B^a^	PipeLine database	January 2010–December 2017	n/a

Abbreviations: DHIMS, district health information management system; DHIS 2, District Health Information System 2; eLMIS, electronic logistics management information system.

a Two countries have been anonymized to enable inclusion of their data in this article.

**TABLE 2.** Inventory Policy on Maximum Months of Stock Holdings for Each Level of the Supply Chain as Determined by Each Country's Supply Chain Design

Level	Burkina Faso	Ethiopia	Ghana	Kenya	Pakistan	Tanzania	Uganda	Country A	Country B
Central medical stores	15 (combined)	5	12	30	14	9	6	12	9
Regional/hubs level		4	6	n/a	n/a	n/a	n/a	6	
District level	5	4	n/a	n/a	3	6	n/a	4	3
SDPs	2	4	3	2	1	n/a	4	2	2
Health posts	n/a	2	n/a	n/a	n/a	n/a	n/a	n/a	n/a
**System total**	**22**	**19**	**21**	**32**	**18**	**15**	**10**	**24**	**14**
**System total used in model**	**22**	**18**	**21**	**30**	**18**	**15**	**10**	**24**	**14**

Abbreviations: n/a, not applicable; SDPs, service delivery points.

#### Procurement and Beginning Balance

Through the Implants Access Program, the research team collected monthly procurement data for all FP2020 countries for levonorgestrel 2-rod, 5-year implant and etonogestrel 1-rod, 3-year implant. Although Sino-implant was procured during this time, available data (from the Reproductive Health Interchange) indicate that procurement volumes were either through social marketing organizations dispensing to markets we did not account for (Ethiopia [DKT] and Pakistan [Marie Stopes International]) or were at percentages below 1% of total procurement (Burkina Faso, Ghana, Kenya, and Uganda).

The procurement data provided by the manufacturers capture the dates of shipments from the manufacturing point but do not include the date of arrival or clearance in the destination country. To obtain this additional information, we reviewed country reports (e.g., procurement plans captured in databases using PipeLine,^13^ a software commonly used for supply planning and monitoring) to identify receipt dates for shipments into countries. If these data were not available in the country supply plan, as is frequently the case for NGO and social marketing organization orders, an estimate was made based on the available country-specific estimated lead times for shipping, evidenced by the above country reports.

Baseline inventory holdings were estimated at the start, with data where available (e.g., PipeLine). If we did not have accurate data for the beginning stock, we assumed no stock on hand for those cases. This assumption is justified because where inventory data were available, the quantities on hand indicated negligible stock levels on implants, especially in comparison with the growth of implant consumption that occurred in the following years. This assumption was also reasonable because our baseline year of 2010–2011 predates the price reduction agreements by several years (signed in 2013) and the other implants (Norplant and Sino-implant) did not represent a significant portion of procurements.

#### Estimates of Users of Implants

One estimate of the number of implants consumed was based on a combination of United Nations population data,^14^ mCPRs, implant share of method mix or implant CPR, and couple year of protection (CYP) conversion factors.^15^ The mCPR, implant share of method mix, and implant CPR were collected for all women (with the exception of Pakistan, for which mCPR and method mix were available only for married women) from DHS and PMA2020 surveys and when used in the below formula can estimate users of implants.^7^^,^^8^ See the Appendix for the data and sources used for each country.

implant users=female population of reproductive age×mCPR×implant share of method mix

Or

implant users=female population of reproductive age×implant CPR

Since our analysis reviewed products procured and available through public health and subsidized channels such as ministries of health and NGO and social marketing organization services, we did not include users who accessed implants from private retail markets. Our estimates for source mix of combined public and NGO sectors also came from DHS (or in the case of Pakistan from the electronic logistics management information system) and are included in the Appendix as well.

#### Converting Users From Demographic Data to Implant Requirements

Multiple methodologies exist to estimate products required to serve a number of users of a particular method for a given period of time. We relied on the conventional method to convert the number of users of a product to quantities of products required for a period of a year used by Reality Check, a tool created by EngenderHealth and widely used by many family planning practitioners to project family planning trends (i.e., CPR and method mix) and the resources required to achieve them. To convert the number of users into estimated number of products required, we used the established concept of CYPs developed by the United States Agency for International Development and respective conversion factors.^15^ CYP factors are an estimate of protection provided by contraceptive methods during a 1-year period and yield an estimate of the average duration of use and contraceptive protection provided per unit of that method and therefore differ from the period of method efficacy. Although CYP factors were not intended to be used for procurement planning, they have been used in most methodologies to estimate product requirements from user data. Since all users would have required an implant at some time, the CYP is used as a proxy to determine the period of time in which they may have received the implant. Because hormonal implants are effective for multiyear periods, the CYP provided is per unit as opposed to units per CYP, as with shorter-acting methods. CYPs for longer-acting methods are also not as long as the full, potential duration of use of the method because they account for early discontinuation and other factors such as wastage. The CYP factors used in our model are presented in Table 3.

**TABLE 3.** Couple Years of Protection^13^ and Conversion Factors

Implant Brand	CYPs/Implant	CYP_CF_
Etonogestrel, 1-rod (Implanon/Implanon NXT)	2.5	1/2.5 = 0.4
Levonorgestrel, 2-rod (Jadelle)	3.8	1/3.8 = 0.26

Abbreviations: CF, conversion factor; CYP, couple year of protection.

We relied on the conventional method to convert the number of users of a product to quantities of products required in a year.

Since demographic survey results do not indicate brand share of implants, we used procurement, consumption mix, and/or forecast assumptions to estimate the share between the 2 brands. The final CYP factor used in the analysis applied country-specific estimated share of the 2 brands. For example, if a country procured, consumed, and/or forecasted a brand mix of 25% levonorgestrel and 75% etonogestrel implants, then the CYP used in the calculations used would be 2.825 as described in this calculation:

(25%×3.8)+(75%×2.5)=2.825,

giving a CYP conversion factor of 1/2.825, or 0.354.

The Reality Check calculation to convert the number of users of a product to quantities of products required for a period of years is as follows^16^:

$$\left(U_{Y2}-U_{Y1}\right)+\left(U_{Y1}\times CYP_{CF}\right)$$

Where:

$$U_{Y1}=Users\;in\;year\;1$$

$$U_{Y2}=Users\;in\;year\;2$$

$$CYP_{CF}=Couple\;year\;protection\;conversion\;factor\left(products\;per\;year\;of\;protection\right)$$

Our calculations required monthly figures; therefore, the above formula was adapted to yield monthly estimates:

$$\left(U_{M2}-U_{M1}\right)+\left(\left(U_{Y1}\times CYP_{CF}\right)\times \frac{1\;year}{12\;months}\right)$$

Where:

$$U_{M1}=Users\;in\;month\;1$$

$$U_{M2}=Users\;in\;month\;2$$

Based on the adapted formula, a CYP of 2.5 for 1-rod, 3-year etonogestrel implant translates to 30 months of protection (with a CYP_CF_ of 1/30 or 0.3̄), and a CYP of 3.8 for the 2-rod, 5-year levonorgestrel implant translates to 45.6 months of protection (with a CYP_CF_ of 1/45.6, or 0.022). This monthly adaptation of the Reality Check calculations results in the same aggregated annual product total as the original Reality Check formula.

Final CYP factors used for each country have not been provided in this article because doing so would divulge confidential market information.

#### Data on Distribution of Implants Directly to Clients

We also collected data on direct-to-client distribution of implants when and where available. Direct-to-client distribution represents quantities of products actually dispensed/used. Typically, distribution of products to clients is tracked through records used at individual SDPs such as health management information systems (tracking the number of women who receive an implant insertion service) or logistics management information systems (tracking the dispensing/consumption of the implant product).

We also collected data on direct-to-client distribution of implants when and where available.

We chose to use actual dispensed-to-client data instead of demographic estimates, explained earlier, as an input to the model when available for answering the first and second research question. This input is described in Table 1.

#### Country Supply Chain Parameters

Country supply chain design and inventory management policies dictate the total system volumes required for the supply chain to respond effectively to variable demand. We reviewed country documents and spoke with country representatives to determine distribution points along the supply chain and respective maximum inventory stocking policies for the 9 countries included in the study. Inventory levels are typically measured in relative terms of demand―that is, months of stock rather than absolute quantities, given that the intent is to be able to assess how long the current stock levels would be expected to last, based on current and future demand. Table 2 documents the maximum months of stock inventory policies for each level of the supply chain in the 9 countries reviewed as well as the system total used in the models.

The total line reflects months of stock that are needed to ensure adequate system inventory levels at all levels of the supply chain, as determined by country supply chain design, and assumes that quantities procured are distributed to distribution points and SDPs as dictated by the supply chain design of the country. The total used in the model for Ethiopia and Kenya deviates from the system totals because not all products flow to the health posts in Ethiopia and maximum system inventory levels are not strictly adhered to in Kenya for procurement purposes. In Pakistan, although some SDPs receive products directly from the central level, those working in the system indicated that the majority of implants are being used in province(s) supplied from the district. Totals used were made in consultation with country implementers familiar with each system.

Although countries typically maintain their inventory levels between predetermined inventory parameters of minimum and maximum stock levels, our model used the system maximum as the benchmark for measuring the surplus or deficit.

All models start in January 2010 (with the exception of Ethiopia and Tanzania, which start in January 2011) and include procurements and estimates of product consumption information through the end of 2017 when data collection for the model was completed. Since we limited further assumptions of implant use past 2017, we used the results of the monthly difference between imputed and filled-to-max inventory up to December 2017, or last survey point available, minus the maximum months of stock required for system inventory. Table 4 represents the period of time used for the results of the analysis for answering research questions 1 and 2.

**TABLE 4.** Period of Time Used to Answer Research Questions 1 and 2

Country	Demographic and/or Dispensed to Client Data Collected	Monthly Model Results Used in Analysis
Burkina Faso	January 2010–December 2017	January 2010–August 2015
Ethiopia	January 2011–January 2016	January 2011–July 2014
Ghana	January 2010–December 2017	January 2010–February 2016
Kenya	January 2010–December 2017	January 2010–June 2015
Tanzania	January 2011–December 2017	January 2011–September 2016
Pakistan	January 2010–December 2017	January 2010–June 2016
Uganda	January 2010–December 2017	January 2010–December 2015
Country A	January 2010–December 2017	January 2010–December 2015
Country B	January 2010–December 2017	January 2010–April 2016

## RESULTS

### Accuracy of Procurement Quantities

**FIGURE 5:**
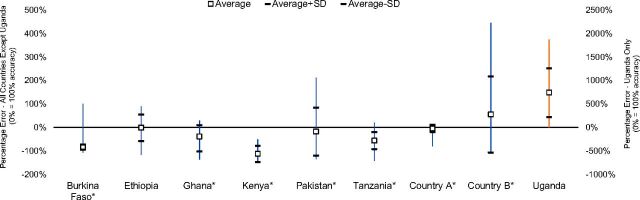
Procurement Quantity Accuracy Based on Maximum Quantities Required to Fill the Supply Chain Abbreviation: SD, standard deviation (average + SD is shown with the top horizontal line while average − SD is shown with the lower horizontal line). Note: Uganda, which appears to have a very high overstocking of implants, is plotted on a separate, secondary y-axis to avoid obscuring the differences for the other countries. *Dispensed-to-client data were incorporated in the analysis.

Three of the 9 countries, Ethiopia, Pakistan, and Country A, on average came closest to accurate procurement over the 5 to 6 years studied, while in 4 countries (Burkina Faso, Ghana, Kenya, and Tanzania), procurement volumes were on average lower than what was required to fill the supply chain to maximum inventory requirement levels. In Country B and Uganda, procurement volumes on average exceeded the need, as shown in Figure 5. Pakistan and Country B had very wide variations in average procurement quantities, while Burkina Faso, Kenya, Tanzania, and Country A had much narrower ranges of procurement quantity accuracy over the period of time reviewed. Figure 2 depicts the results of how accurately countries were able to procure implants through 4 to 6 years (referenced in Table 4) and compares maximum inventory needs of each country's supply chain based on actual procurement to what should have been procured (filled-to-max inventory procurement quantity) for the estimated demand. Countries with asterisks in Figure 5 have actual dispensed-to-client data incorporated in the analysis. Any country that crosses the 0% line had periods of time when it had accurate stock, based on the analysis.

3 countries had accurate procurement, while 4 countries had volumes below needs and 2 countries had excessive volumes.

To interpret the results in Figure 5, 0% procurement error indicates that procurement quantities on average matched the need of the supply chain perfectly. Percentages below 0% indicate that procurement quantities were on average insufficient to fill the supply chain to maximum system inventory levels to meet the estimated demand. In contrast, percentages above 0% indicate that procurement quantities on average exceeded what was needed to fill the supply chain with system maximum inventory to meet the estimated demand. For example, in Tanzania procurement volumes for this period were 56% lower than what would have been required to maintain maximum inventory levels; however, this result may not mean that Tanzania was understocked, or had procurement quantities below what was required for maintaining minimum inventory levels, because this possibility was not included in this analysis.

The results for Uganda were an anomaly. The data appeared to show on average a very high overstocking of implants. However, upon further review, all countries but Uganda have access to some level of dispensed-to-client data. These countries also use such consumption data to inform forecasting. Uganda, on the other hand, historically has relied heavily on demographic methodology for forecasting demand. A situation in which demographic estimates cannot be compared to dispensed or issued data may result in overstocking or the *appearance* of overstocking because we do not have accurate data on dispensed-to-client data of implants in Uganda.

Had there been no actual dispensed-to-client data available, forcing us to use only *lower* demographic estimated implant consumption in the model (see results to research question 3), procurement quantity accuracy would actually *appear* to increase. The same procurement volumes would *better* fill a system to max levels when there appears to be a lower demand. Table 5 presents the comparison of these 2 results with all but one country showing a lesser degree of under-procurement or even over-procurement when using our demographic estimates in the model.

Simply applying a percentage to demand to account for system inventory needs is likely to produce inaccurate procurement quantities.

**TABLE 5.** Comparison of Procurement Quantity Accuracy Figures Based on Use of Dispensed-to-Client Data^a^

Country	Average Procurement Quantity Error	
	No Dispensed-to-Client Data Used in Model (Only Demographic Estimates Used)	Dispensed-to-Client Data Used, if Available, in Model
Burkina Faso	**−29%**	−83%
Ghana	**−23%**	−39%
Kenya	**−111%**	−112%
Tanzania	**51%**	−56%
Country A	**−7%**	55%
Country B	112%	**−4%**

a Bolded figures indicate the appearance of more accurate result, and negative figures represent under-procurement.

This increased likelihood of showing over-procurement when using demographic data for demand estimates may be why Uganda appears to be overstocked in Figure 5. Had there been dispensed-to-client data (to show higher consumption than demographic estimates), the system may in reality not have been overstocked.

### Standard Factor for Relating Consumption Data to Procurement Volumes

**FIGURE 6:**
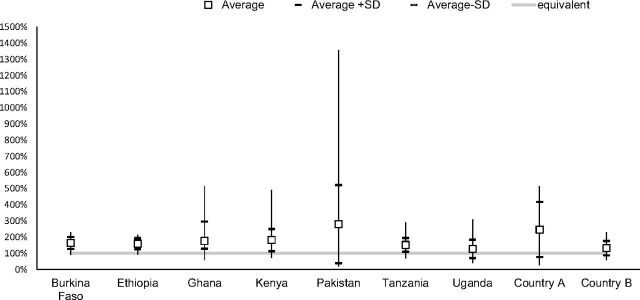
Inventory Needs in a Month as a Percentage of Consumption in the Same Month Abbreviation: SD, standard deviation.

Our results also demonstrated that there was no standard factor for relating inventory quantities to consumption rates across countries or even within a country through time as the program demand changes. Inventory needs in a month as a percentage of consumption in the same month varied from 18% to 1,356% and averaged about 180% for the countries and periods evaluated, as shown in Figure 6, almost double what was consumed. We believe that these results do not just pertain to implants only but to any product that does not have stable demand. Therefore, simply applying a percentage to demand to account for system inventory needs is likely to produce inaccurate procurement quantities.

No standard factor existed for relating inventory quantities to consumption rates across countries or even within a country through time.

The data also showed that inventory levels and related procurement volumes do not have a consistent relationship to consumption volumes. The range of percentages is explained by context-specific factors that influence the proportion of consumption/demand that should be in inventory (i.e., procured) and vary based on system design parameters and rates of growth or decline in consumption. The results also reflect the rapid rates of scale-up associated with the implant programs in countries. The country with the widest range, Pakistan, also had the highest percentage growth in implant users, increasing from 6 users in the first month of the analysis to 12,370 in a peak month.

### Accuracy of Demographic Estimates

**FIGURE 7:**
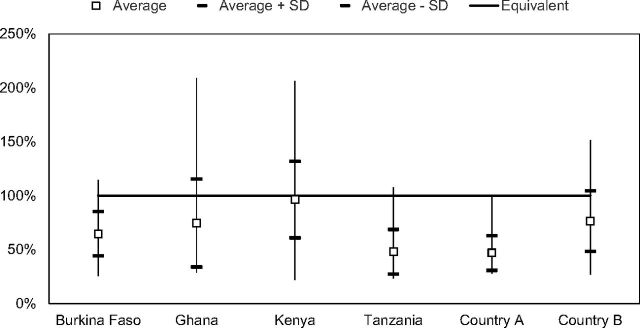
Calculated Consumption (Using Demographic Estimates) as a Percentage of Dispensed-to-User Data Abbreviation: SD, standard deviation.

Comparison of demographic estimates of consumption to dispensed-to-client data for the 6 countries for which both sets were available revealed that demographic estimates (using the Reality Check methodology) were on average lower than the dispensed-to-client consumption based on data from *all* 6 countries. Comparisons were available for 435 months (over 36 years) of data. For each country, the average consumption of implant forecasts estimated using demographic estimates as a percentage of dispensed-to-client data were below 100% and ranged from 47% to 96%, yielding forecast errors in using demographic estimates that ranged from 4% to 53%. The averages, range, and standard deviations were also calculated for each country and are provided in Figure 7.

To interpret Figure 7, in Tanzania, the estimated forecasts using demographic estimates on average represented only 48% of the number of implants dispensed to clients based on the electronic logistics management information system data reported at facilities; the average forecast error would have been 52% had forecasts been made using demographic estimates.

The findings for question 3, indicate that demographic estimates, using the Reality Check methodology, underestimate dispensed-to-client consumption of implants. Although this study did not evaluate the reasons for the discrepancy, studies evaluating the validity of using CYP conversion factors have highlighted challenges, particularly for condoms and long-acting contraceptives.^17^^,^^18^ This underestimate of implant demands using demographic estimates has implications for the use of this demographic methodology (including CYPs) for forecasting consumption for contraceptive implants.

Demographic estimates underestimate dispensed-to-client consumption of implants.

### Model Evaluation

To validate the results of the model, the outputs of the imputed ending inventory balance were compared to reported total system-wide full inventory in Burkina Faso, one country for which these data were available. Burkina Faso reports system inventory (central, regional, and SDP combined) in its PipeLine^13^ database and routinely updates the stock on hand at the time of physical inventory. The recorded system inventory data were available for the following periods: January 2010, January 2011, January 2012, July 2012, January 2013, July 2013, January 2014, July 2014, January 2015, July 2015, January 2016, and July 2016.

**FIGURE 8:**
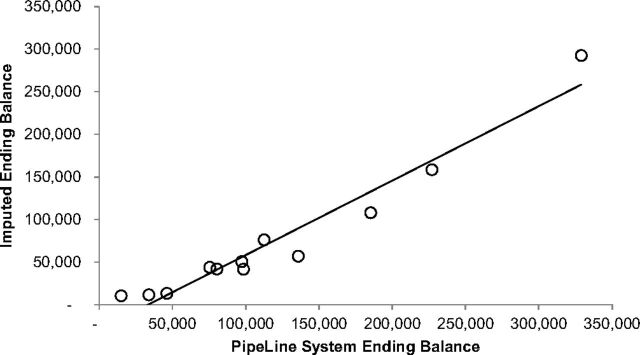
Burkina Faso Scatter Plot of PipeLine Reported System Inventory and Imputed Ending Balance for the Same Reporting Months

We compared these reported system inventories to the model output of imputed ending inventory for the same months and plotted the results in Figure 8. The resulting Pearson correlation coefficient of the 2 datasets is 0.97061, indicating a high degree of relationship between the 2 data sets.

Based on this analysis, we feel confident that the model outputs align with imputed stock-on-hand information on the ground. Similar quality data to validate model outputs were unavailable for other countries.

## DISCUSSION

Our results show that the significant investments in procurement quantities for the rapidly growing implant programs in the 9 countries were justified based on consumption rates. The results also show that the investments were likely an important enabler for countries to achieve the high insertion rates for this new method and for implants to achieve a growing proportion of mCPR for the period reviewed. Further, the results suggest that most of the countries included in this evaluation historically have not been ordering quantities that meet their systems' maximum inventory needs, given the length of their supply chains and the rate of growth in use of implants. However, countries that had estimated quantities of inventory below the system filled-to-max inventory levels should not be interpreted as having insufficient quantities or quantities below the system minimum stock levels because this research did not attempt to answer that question. The analysis also did not attempt to assess overstocking above maximum inventory levels within the in-country pipeline or by distribution level.

The significant investments in procurement quantities for implants in the 9 countries were justified based on consumption rates.

Study results also suggest that large stocks of product accumulating unused in national or subnational warehouses do not explain the difference between quantities procured and insertion rates. Rather, the variation in the inventory-to-consumption ratio of 18% to 1,356% demonstrated in Figure 6 reflects the variation in efficiency of each country supply chain in moving contraceptives from ports of arrival through the in-country system to end users. With each additional level that the product has to travel through and with limitations in storage and distribution infrastructure, varying quantities of inventory are needed for each country to ensure the pipeline is filled at each level so that facilities can be resupplied regularly and clients can access an implant whenever they need one.

In addition, our results demonstrate that calculated consumption using demographic estimates (with the Reality Check methodology) as a percentage of dispensed-to-client data was below 100% in all 6 countries for which both types of data were available, and ranged from 47% to 96%. These forecast errors in using demographic data ranged from 4% to 53% and are likely one reason for underestimates, given that many programs use demographic estimates as the primary forecasting methodology.

Best practice recommends that countries use multiple methodologies of forecasting demand, review the strengths and weakness of each methodology, and compare results to select final forecasts outputs (either as a blended or soundest forecast).^10^^,^^12^ Yet some national programs may not be able to forecast using multiple methodologies and sources of data for 2 reasons. The first is the limited visibility and availability of consumption (dispensed to client and/or issues) and services data from routine information systems. When systems do not provide sufficiently complete or credible data, program managers may rely heavily on demographic data and estimates for forecasting. The second reason for not using multiple methodologies and sources of data is that the relevant skills and practices may not be widespread in many countries, even when data are available. Our results indicate that countries that rely solely on demographic data and use the demographic forecasting methodology (using the same methodology as in this analysis) may routinely under-forecast contraceptive implants and be unable to completely fill their supply chain with sufficient inventory.

Countries that rely solely on demographic data and use the demographic forecasting methodology may be unable to maintain sufficient inventory of implants.

It is important to consider the results of this overall analysis in the context of general supply chain management principles and as governments, donors, and partners consider investments in supply chain strengthening. As with any forecast, accuracy is never 100% and errors are typical. Inventory must be available in advance of service to make product choice and increasing mCPR possible; in this scenario, if product demand is increasing, order quantities will continue to increase and the supply chain will not be “filled” until the growth in demand levels off to zero. This advanced time is equal to the length of the supply chain at maximum system inventory requirements. Therefore, based on demand trends for implants, large quantities of inventory (and respective procurements) are needed to fill the supply chain and should not be considered excess stock.

Another relevant supply chain management concept to consider in relation to this study is cycle (or customer) service levels―the ability to meet client demand on the day of visit with the product desired. Service levels below 100% for any given period mean that not all demand was satisfied and there was a stock-out. Contraceptive stock-outs are one of the primary and recurring challenges that affect many countries and jeopardize women's reliable access to family planning products. Stock-outs also pose a major risk to the ambitious goals of FP2020. While family planning programs have placed increased emphasis on ensuring no stock-outs, it is important to recognize that realistically, reaching 100% customer service level (or no stock-outs ever) is not achievable and requires quantities of inventory far in excess than resources allow.^11^^,^^12^ That said, ensuring that sufficient inventory is available (including sufficient safety stock at points of resupply) and supply chains can be responsive to any sudden changes in demand is the best way to minimize both the frequency and duration of stock-outs.

To decrease inventory and yet continue to provide the same or greater level of service, supply chains need to be more efficient and/or responsive. With changes to supply chain design, it is possible to increase inventory turns while still keeping customer service levels high and to reach goals. Several levers can be applied to achieve greater efficiency, including decreasing the number of distribution points or lead times between distribution points; increasing review and reorder frequency; holding stock at lower levels of the supply chain; and finally, reducing the uncertainty of lead times and demand variability.^11^ It is important to note that responsible redesign of supply chains requires understanding of all costs in the supply chain, not just inventory holding costs, because making changes without understanding all costs may increase total system costs.

Responsible redesign of supply chains requires understanding of all costs in the supply chain.

The results also emphasize the importance of establishing and strengthening electronic logistics management systems that collect supply chain data and the use of these data for operational and management decisions—including for robust forecasting and quantification—to improve performance. Understanding true demand at the last link of the supply chain (e.g., at the SDP) is essential for managing supply throughout the chain. Without this information, supply chain managers have limited ability to increase performance and efficiency of their systems, which ultimately limits women's access to family planning and their choice of method.

This study also raised a few considerations about the use of CYP when used with the Reality Check methodology to convert number of users to quantity of products, particularly for long-acting methods. CYP is widely used to estimate the number of couples protected from pregnancy based on the quantities of family planning contraceptives distributed, and it allows donors and programs to estimate the impact of the contraceptives provided.^19^ Although not developed for this purpose, the conversion factor has been widely used in demographic forecasting to convert number of users to products required, a methodology described earlier. Recent studies evaluating the validity of using the conversion factors to determine products required have highlighted challenges, particularly for condoms and long-acting contraceptives. This challenge likely reflects inadequate data on quantities typically used (as in the case of condoms) and duration of actual use (as in the case of long-acting reversible contraceptives).^17^^,^^18^ Our results indicate that estimates using demographic data with the Reality Check methodology and CYPs set consumption for implants too low. Therefore, other methodologies, such as the Marie Stopes International Impact 2 model, should be tested to determine if they present better options for determining product needs from users.^20^

### Assumptions and Limitations

The model uses a number of assumptions about the data and relationships. The system filled-to-max inventory procurement quantity assumes that the country has no resource limitations and that product is available at the top of the supply chain (procured) to flow through the system at the right time. The model also assumes that products flow through the in-country system and are supplied downstream to distribution points and SDPs as the system design dictates.

Another assumption is that forecasts and supply plans are developed solely at the central level and are accurate. Assuming forecast error of any percentage, a norm with all forecasts and supply chains, would require additional quantities of inventory to account for this variance in demand. Further still, if each level of the system was doing its own forecast to determine its demand, additional inventory would be needed at each point of the supply chain to account for the further uncertainty in demand at each level.

Although directly measuring dispensed-to-client data quality was not possible, we reviewed data received to ensure there were no obvious data entry or other errors. We had access to data from other countries in addition to those included, but used peer judgment of quality to only include countries that provided better quality dispensed-to-user data in the analysis. We also assume that the supply chains are as described in Table 2 and that countries are following supply chain designs as described. Lastly, our model assumes that clients consume all products. The model does not account for wastage and/or products used for training, and it assumes no loss or damage of products, which would need to be removed from the system and deducted from stock levels before reaching a client.

## CONCLUSION

This research should provide assurances that rapid increases in procurement quantities of implants for countries where we have data have not resulted in system overstocks to date. Our results should also reinforce the idea that the relationship between procurement quantities and consumption levels cannot be accurately assessed without understanding the country supply chain, inventory control parameters, and current and future demand. Further, the need to procure large quantities of products well in advance of when they are needed may be reasonable, although it does raise questions about opportunities to redesign supply chains such that inventory (and related holding costs) can be reduced and what the cost and service level benefits of a redesign might be.

This study also provides ideas for future research. First, a future research study could explore other methodologies for demographic estimates and the validity of using CYP factors when converting users to number of products used, particularly for long-acting methods such as contraceptive implants. Second, future research could evaluate countries with longer, multilevel supply chains to cost out the supply chain current state, including the cost of holding inventory, and potential alternative scenarios to determine if supply chain redesign (reducing levels, increasing order frequency) could help reduce the inventory required to meet demand while also minimizing total systems costs.

## Supplementary Material

19-00017-Akhlaghi-Supplement1.pdf

19-00017-Akhlaghi-Supplement2.pdf

## Notes

**Peer Reviewed**

**Funding:** Bill & Melinda Gates Foundation

## Appendix

**APPENDIX:** Demographic Data Used in Estimation of Implant Users

Country	Data Source	All Women			Public-Sector and NGO Source Mix
		mCPR	Implant CPR	Implant Share of Method Mix	
Burkina Faso	DHS 2010	14.3	2.9		92
	PMA-R1 2014	15.7		43.4	
	PMA-R2 2015	18.6		34.9	
	PMA-R3 2016	21.5		41.9	
	PMA-R4 2017	21.9		43.4	
Ethiopia	DHS 2011	18.7	2.3		94
	PMA-R1 2014	22.5		15.6	
	PMA-R2 2014	23.8		20.4	
	PMA-R3 2015	25.6		20.2	
	PMA-R4 2016	26.5		22.9	
	DHS 2016	24.9	5.7		96.8
Ghana	DHS 2008	13.5	0.7		85.4
	PMA-R1 2013	14.3		13.3	
	PMA-R2 2014	14.6		14.1	
	DHS 2014	18.2	3.7		94.1
	PMA-R3 2014	18.1		12.6	
	PMA-R4 2015	23.4		14.9	
	PMA-R5 2016	21.7		18.3	
	PMA-R6 2016	21.3		23.4	
Kenya	DHS 2008	28	1.3		81
	DHS 2014	39.1	7.1		82.1
	PMA-R1 2014	42.5		18.3	
	PMA-R2 2014	40.3		18	
	PMA-R3v2015	46.5		21.1	
	PMA-R4 2016	46		23.9	
	PMA-R5 2016	44.2		27.6	
	PMA-R6 2017				
Pakistan	DHS 2006-2007	21.7	0.1		eLMIS data by month
	DHS 2012-2013	26.1	0		
Tanzania	DHS 2010	23.6	1.8		94.8
	DHS 2015-2016	27.1	5.6		97.8
Uganda	DHS 2011	20.7	1.9		87.5
	PMA-R1 2014	21		11.9	
	PMA-R2 2015	26.2		12.5	
	PMA-R3 2015	25.9		14.3	
	PMA-R4 2016	27.5		13.1	
	DHS 2016	27.3	4.7		84
Country A	DHS 2006	4.5	0		n/a (100% used)
	DHS 2012	11	0.3		n/a (100% used)
	PMA-R2 2016	12.6		17.6	
Country B	DHS 2007-2008	16.3	0.9		
	DHS 2010	25.2	3.6		94.8
	DHS 2014-15	27.8	4.7		92

Abbreviations: CPR, contraceptive prevalence rate; DHS, Demographic and Health Survey; eLMIS, electronic logistics management information system; mCPR, modern contraceptive prevalence rate; n/a, not applicable; PMA, Performance Monitoring and Accountability.
