# Supplementary material for: Are Procured Quantities of Implants Adequate and Appropriate? Modeling Procurement, Inventory, and Consumption of Contraceptive Implants During Rapid Uptake
Source: Glob Health Sci Pract. 2019 Jun 24;7(2):240–57. doi: 10.9745/GHSP-D-19-00017 (PMC6641805; doi:10.9745/GHSP-D-19-00017)
Supplement: 19-00017-Akhlaghi-Supplement1.pdf [file 19-00017-Akhlaghi-Supplement1.pdf]

Akhlaghi L, Heaton A, Chandani Y. Are procured quantities of implants adequate and appropriate? Modeling procurement, inventory, and consumption of contraceptive implants during rapid uptake. *Glob Health Sci Pract*. 2019;7(2). https://doi.org/10.9745/GHSP-D-19-00017

SUPPLEMENT 1. Calculation of System Imputed, Filled-to-Max Inventory, and Percentage Difference—Ethiopia as an Example

We estimated the imputed system inventory by subtracting use and adding shipments to the beginning inventory and continuing this calculation for each month of the model.

$$Imputed\ Inventory_{Mo\ 2} = Imputed\ Inventory_{Mo\ 1} - Implant\ Use_{Mo\ 2} + Procured\ Quantities_{Mo\ 2}$$

Where:

Mo 1: Month 1

Mo 2: Month 2

Table A1. Calculation of System Imputed Inventory

	…	Mo 1	Mo 2	Mo 3	Mo 4	Mo 5	Mo 6	Mo 7	Mo 8	Mo 9	Mo 10	Mo 11	Mo 12	Mo 13	Mo 14	Mo 15	Mo 16	Mo 17	Mo 18	…
Implant use (demographic estimate)		24,328	24,417	24,507	24,596	24,685	24,775	24,865	24,955	25,045	25,136	25,226	25,317	28,726	28,819	28,911	29,004	29,098	29,191	
Procured quantities			0	250,048	112,736	70,016	155,616	119,000	0	58,600	50,000	50,000	50,000	0	75,008	50,000	0	0	0	
Imputed inventory		218,723	194,305	419,847	507,987	553,317	684,158	778,293	753,338	786,893	811,757	836,531	861,215	832,489	878,678	899,767	870,763	841,665	812,474	

Note: Country models span 72–84 months. A representative period of 18 months for 1 country is provided here to illustrate the example due to space constraints.

Figure 1 (included in the main article) is a graphical representation of the imputed inventory in Table A1.

To determine the quantity of inventory required to fill the system to the maximum levels defined by the country-specific system design, we sum the implants used for the length of time equivalent to the system max. For example, if the system max is 18 months of stock, the system maximum inventory would be equal to the total implants used from month 1 through month 18. If the system max of a country was 12 or 22 months, the model sums the implant use for the respective number of months.

$$Inventory\ required\ to\ fill\ system\ to\ max_{Mo\ 1} = \sum_{i=Mo\ 1}^{system\ max} Implant\ use$$

Where:

$\sum_{i=Mo\ 1}^{system\ max} Implant\ use$ = Sum of implants used from month 1 to the last month required for the length of the system max

Akhlaghi L, Heaton A, Chandani Y. Are procured quantities of implants adequate and appropriate? Modeling procurement, inventory, and consumption of contraceptive implants during rapid uptake. *Glob Health Sci Pract*. 2019;7(2). https://doi.org/10.9745/GHSP-D-19-00017

Table A2. Calculation of System Filled-to-Max Inventory

	…	Mo 1	Mo 2	Mo 3	Mo 4	Mo 5	Mo 6	Mo 7	Mo 8	Mo 9	Mo 10	Mo 11	Mo 12	Mo 13	Mo 14	Mo 15	Mo 16	Mo 17	Mo 18	…
Implant use		24,328	24,417	24,507	24,596	24,685	24,775	24,865	24,955	25,045	25,136	25,226	25,317	28,726	28,819	28,911	29,004	29,098	29,191	
Length of pipeline (months)	18	System max used in Ethiopia model																		
Inventory required to fill system to max		471,601	476,557	481,517	486,483	491,453	496,427	501,406	510,063	518,726	527,396	536,072	562,527	589,200	612,768	636,551	660,547	684,758	709,184	

Figure 2 (included in the main article) is a graphical representation of the filled to max inventory in Table A2.

Using the below calculation, we would be able to determine the percentage difference for each month and answer our first research question.

$$\frac{\text{"imputed" inventory} - \text{"filled to max" inventory}}{\text{"filled to max" inventory}} = Percentage\ difference$$

Table A3. Calculation of Percentage Difference of Imputed Inventory and Filled-to-Max Inventory

	…	Mo 1	Mo 2	Mo 3	Mo 4	Mo 5	Mo 6	Mo 7	Mo 8	Mo 9	Mo 10	Mo 11	Mo 12	Mo 13	Mo 14	Mo 15	Mo 16	Mo 17	Mo 18	…
Imputed inventory	…	218,723	194,305	419,847	507,987	553,317	684,158	778,293	753,338	786,893	811,757	836,531	861,215	832,489	878,678	899,767	870,763	841,665	812,474	…
Inventory required to fill system to max	…	471,601	476,557	481,517	486,483	491,453	496,427	501,406	510,063	518,726	527,396	536,072	562,527	589,200	612,768	636,551	660,547	684,758	709,184	…
Deficit or surplus	…	-252,878	-282,251	-61,671	21,504	61,865	187,731	276,887	243,275	268,167	284,361	300,459	298,687	243,289	265,910	263,216	210,215	156,907	103,290	…
Percentage difference	…	-54%	-59%	-13%	4%	13%	38%	55%	48%	52%	54%	56%	53%	41%	43%	41%	32%	23%	15%	…

Figure 3 (included in the main article) is a graphical representation of the deficit or surplus in Table A3.

The percentage differences for each month of the model were used to calculate the averages and standard deviations used in the results to answer question 1 (Figure 5 in the main article).
