# Supplementary material for: Are Procured Quantities of Implants Adequate and Appropriate? Modeling Procurement, Inventory, and Consumption of Contraceptive Implants During Rapid Uptake
Source: Glob Health Sci Pract. 2019 Jun 24;7(2):240–57. doi: 10.9745/GHSP-D-19-00017 (PMC6641805; doi:10.9745/GHSP-D-19-00017)
Supplement: 19-00017-Akhlaghi-Supplement2.pdf [file 19-00017-Akhlaghi-Supplement2.pdf]

Akhlaghi L, Heaton A, Chandani Y. Are procured quantities of implants adequate and appropriate? Modeling procurement, inventory, and consumption of contraceptive implants during rapid uptake. *Glob Health Sci Pract*. 2019;7(2). https://doi.org/10.9745/GHSP-D-19-00017

SUPPLEMENT 2. Inventory Needs in a Month as a Percentage of Consumption

When demand for a product is stable and inventory is filled, what enters the system in month one would equal the amount leaving the system at the end of the supply chain. For example, in Ethiopia if the inventory is filled and demand is stable, the quantity used in month one would equal the quantity used in month 18. Therefore, the quantity procured and needed in month one would be equal to use of implants in month one and should also equal the last month of the system max in order to keep inventory filled to max. The inventory needs as a percentage of consumption in a filled, stable system would therefore be 100%, using the following calculation.

$$\text{Inventory needs as a percentage of consumption} = \frac{Implant\ Use_{Mo\ n}}{Implant\ Use_{Mo\ n+system\ max-1}}$$

Where:

n= month number

$$\text{In the Ethiopia example, this would be} = \frac{Implant\ Use_{\ Mo\ 1}}{Implant\ Use_{\ Mo\ 18}}$$

To determine if there is a standard factor that can be applied to consumption data to predict procurement volumes when demand is not stable and country systems differ in length of system max, we applied this calculation to the months available in our country models. The percentages for each month of the model were used to calculate the averages and standard deviations used in the results to answer question 2 (Figure 6 in the main article).
